# Efficient access to β*-*vinylporphyrin derivatives via palladium cross coupling of β-bromoporphyrins with *N*-tosylhydrazones

**DOI:** 10.3762/bjoc.13.22

**Published:** 2017-01-30

**Authors:** Vinicius R Campos, Ana T P C Gomes, Anna C Cunha, Maria da Graça P M S Neves, Vitor F Ferreira, José A S Cavaleiro

**Affiliations:** 1 Departamento de Química Orgânica, Instituto de Química, Universidade Federal Fluminense, 24020-150 Niterói, RJ, Brazil; 2 QOPNA and Department of Chemistry, University of Aveiro, 3810-193 Aveiro, Portugal

**Keywords:** *N*-tosylhydrazones, Pd-catalyzed cross coupling, porphyrins

## Abstract

This work describes a new approach to obtain new β-vinylporphyrin derivatives through palladium-catalyzed cross-coupling reaction of 2-bromo-5,10,15,20-tetraphenylporphyrinatozinc(II) with *N*-tosylhydrazones. This is the first report of the use of such synthetic methodology in porphyrin chemistry allowing the synthesis of new derivatives, containing β-arylvinyl substituents.

## Introduction

Porphyrin-type heterocycles are well known for their special role in several scientific fields, including medicine [1–3] , supramolecular chemistry [4–6] and catalysis [7–10]; their functionalization became a target for several research groups [11–13]. In particular, the insertion of functional groups into the macrocyclic core merits considerable attention due to the diversity of structural features that can be induced in the porphyrin structure [14–16].

Alkenyl-type moieties are examples of versatile functional groups which are present in several biologically active natural porphyrin derivatives or are substituents that can be introduced into the macrocycles, affording in such way important intermediates for different synthetic strategies [4,17–18].

**Scheme 1:**
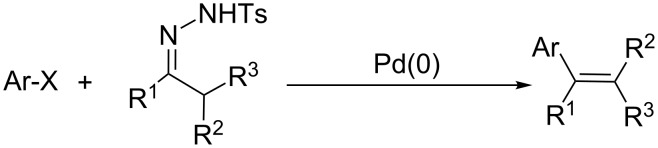
Schematic representation of palladium-catalyzed cross-coupling reaction between aryl halides and *N*-tosylhydrazones.

There are methods available allowing the insertion of alkenyl groups into porphyrin macrocycles (e.g., Heck reaction [4], metathesis [7,19–21], Wittig reaction [22–23]). However, palladium-catalyzed cross-coupling reaction between *N*-tosylhydrazones and aryl halides seems to be a powerful method to synthesize new alkene-type derivatives [24–25]. This methodology allowed the development of novel protocols enabling transformations that are difficult to achieve with other reactions and include other important features – stoichiometric organometallic reagents are not required for the coupling reaction and di- and trisubstituted olefins can be prepared with high stereoselectivity. In fact, *N*-tosylhydrazones used in this methodology can be easily prepared from carbonyl compounds (Scheme 1), as it has been well demonstrated by Barluenga and co-workers [11,26–27].

This methodology allowed the development of novel protocols enabling transformations that are difficult to achieve with other reactions and include other important features as: stoichiometric organometallic reagents are not required for the coupling reaction.

**Scheme 2:**
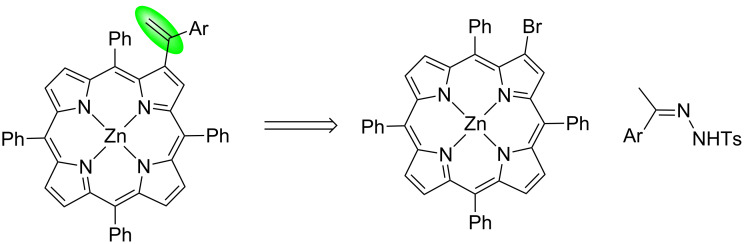
A retrosynthetic scheme for the synthesis of β-alkenyl-type porphyrin derivatives from the Zn(II) complex of β-bromo-*meso*-tetraphenylporphyrin and *N*-tosylhydrazones.

Interestingly, this methodology has never been applied to the synthesis of porphyrin derivatives containing alkenyl groups at β-pyrrolic positions. Having this in mind and following our interest in the development of synthetic approaches to prepare novel porphyrin derivatives [28–30], we decided to explore the palladium-catalyzed cross-coupling reaction of *meso*-tetraphenyl-β-bromoporphyrin using *N*-tosylhydrazone derivatives with different electronic features for the preparation of new β-alkenyl-type porphyrin derivatives. This approach can provide interesting compounds with potential biological properties or might become important templates for further functionalization procedures (Scheme 2).

## Results and Discussion

**Scheme 3:**
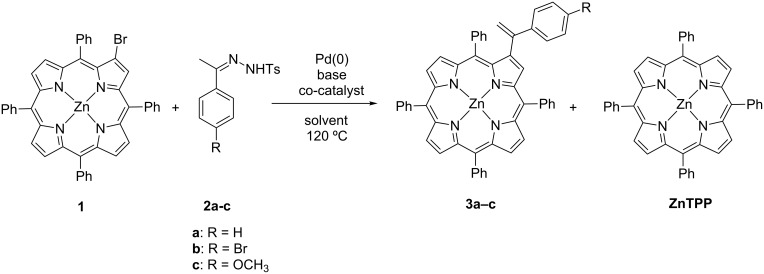
Palladium catalysed cross-coupling reactions between β-brominated porphyrin **1** and *N*-tosylhydrazones **2a–c**.

For this work 2-bromo-5,10,15,20-tetraphenylporphyrinatozinc(II) (**1**) was used as the aryl halide and also the easily accessible *N*-tosylhydrazones **2a–c** (Scheme 3). Both substrates were prepared by following literature procedures; the halide partner by controlled bromination of 5,10,15,20-tetraphenylporphyrin (TPP) with *N*-bromosuccinimide followed by metallation with Zn(AcO)_2_ [31–32] and the tosylhydrazones **2a–c** by reaction of the adequate ketones with tosylhydrazines [33]. The use of the porphyrin zinc complex avoids the complexation of the tetrapyrrolic macrocycle with Pd(II) formed during the catalytic cycle, and in this way prevents the loss of the catalyst.

In the first experiments, the reactions between 2-bromo-5,10,15,20-tetraphenylporphyrinatozinc(II) (**1**) and the *N*-tosylhydrazone derivatives **2a–c** were performed in the presence of Pd(PPh_3_)_4_ as catalyst, KO*t*-Bu as base and 2-dicyclohexylphosphino-2’,4’,6’-triisopropylbiphenyl (Xphos) as ligand. All reactions were carried out in a sealed Schlenk tube in toluene and dioxane at 120 °C under conventional heating and anhydrous air-free conditions (Scheme 3) and stopped when the TLC controls showed no more evolution of the reaction progress. Having in mind the possible self-reaction of tosylhydrazone **2b**, a process competing with the required reaction of **2b** with bromoporphyrin, it was decided to use an excess of the tosylhydrazone.

The results obtained after the work up and chromatographic purification are summarized in Scheme 3 and Table 1 (entries 1, 2 and 3). Under these conditions, the expected β-substituted vinylporphyrin derivatives **3a–c** were obtained in 22–25% yields. No significant differences between the reactivity of the used *N*-tosylhydrazones **2a–c** were observed. The major compounds isolated in these conditions were the starting porphyrin **1** and the zinc(II) complex of TPP (**ZnTPP**) resulting from the debromination of porphyrin **1**.

**Table 1:** Results obtained in the palladium-catalyzed cross-coupling reactions between β-brominated porphyrin **1** with *N*-tosylhydrazones **2a–c**.

Conventional heating								

Entry	*N*-Tosyl- hydrazone **2**	Base	Co-catalyst	Solvent	Reaction time	η **3** (%)	% **1** recov.	% **ZnTPP**

1	**a**	KO*t*-Bu	XPhos	toluene/ dioxane	24 h	23	30	35
2	**b**	KO*t*-Bu	XPhos	toluene/ dioxane	24 h	22	28	30
3	**c**	KO*t*-Bu	XPhos	toluene/ dioxane	24 h	25	31	32
4	**a**	Cs_2_CO_3_	Et_4_NBr	toluene/ DMF	4 h	89	<1	–
5	**b**	Cs_2_CO_3_	Et_4_NBr	toluene/ DMF	4 h	82	10	–
6	**c**	Cs_2_CO_3_	Et_4_NBr	toluene/ DMF	4 h	86	9	–

MW irradiation								

Entry	*N*-Tosyl- hydrazone **2**	Base	Co-catalyst	Solvent	Reaction time	η **3** (%)	% **1** recov.	% **ZnTPP**

7	**a**	Cs_2_CO_3_	Et_4_NBr	toluene/ DMF	5 min	69	15	–
8	**b**	Cs_2_CO_3_	Et_4_NBr	toluene/ DMF	5 min	59	22	–
9	**c**	Cs_2_CO_3_	Et_4_NBr	toluene/ DMF	5 min	64	18	–

The reductive removal of the halo moiety has been already described in the literature for other palladium-catalyzed cross-coupling reactions [34–36], namely in some reactions involving halogenated porphyrin derivatives [37]. Presumably in this particular case the lack of efficiency of the catalytic system is probably responsible for this secondary reaction. Attempts to improve the efficiency of the process just by changing the catalyst to tris(dibenzylideneacetone)dipalladium(0), [Pd_2_(dba)_3_], and the base to lithium *tert*-butoxide, led to the same low efficiency. This fact prompted us to select the catalytic system described by Wang et al. [38]. In their work the authors reported an efficient methodology for the palladium-catalyzed cross-coupling reaction of electron-deficient aryl halides with aryl *N*-tosylhydrazones. In such a way, under these new conditions, the reactions between β-bromonated porphyrin **1** and *N*-tosylhydrazone derivatives **2a–c** were performed in the presence of Pd(PPh_3_)_4_ (5 mol %), 3 equivalents of Cs_2_CO_3_ and 20 mol % of Et_4_NBr as the co-catalyst, using as solvent a mixture of toluene and DMF at 120 °C (Table 1, entries 4, 5 and 6). The results show that under these conditions and after 4 h of reaction, the β-alkenylporphyrin derivatives **3a–c** were obtained in remarkable yields (86–89%). Under these conditions the desired products were obtained in much better yields and in shorter reaction times. Additionally, the presence of **ZnTPP** was not detected in these circumstances and porphyrin **1** was recovered in low amounts (between 0 and 10%). These results confirmed that the improvement of the catalytic efficiency is responsible for diminishing the competitive process that induces the debromination of porphyrin **1**.

Still looking for lower reaction times, we decided to use the latter reactional conditions but under microwave (MW) irradiation (Table 1, entries 7, 8 and 9). The reactions were carried out under 200 W for 5 minutes at 120 °C. The results obtained show that, under MW radiation, β-alkenylporphyrin derivatives **3a–c** were obtained in reasonable yields (between 59 and 69%) and it was possible to recover ca. 20% of porphyrin **1**. The use of MW irradiation did not bring any improvement in the present porphyrin derivatives syntheses.

We believe that the mechanism of the formation of the β-alkenylporphyrin derivatives **3a–c** follows the one reported by Barluenga [13,39–42].

### Structural characterization of the new β-vinylporphyrin derivatives **3a–c**

The structures of the new β-vinylporphyrin derivatives **3a–c** were assigned on the basis of their ^1^H and ^13^C NMR spectra and their molecular formulae were confirmed by HRMS–ESI^+^. 2D NMR spectra [COSY, HMBC, HSQC] were also obtained to unequivocally identify proton and carbon resonances (see Supporting Information File 1).

The ^1^H NMR spectra of porphyrin derivatives **3a–c** show the same type of profile; the principal differences are seen in the resonances of the protons and carbon centres of the aryl group inserted by the palladium-catalyzed coupling reaction.

**Figure 1:**
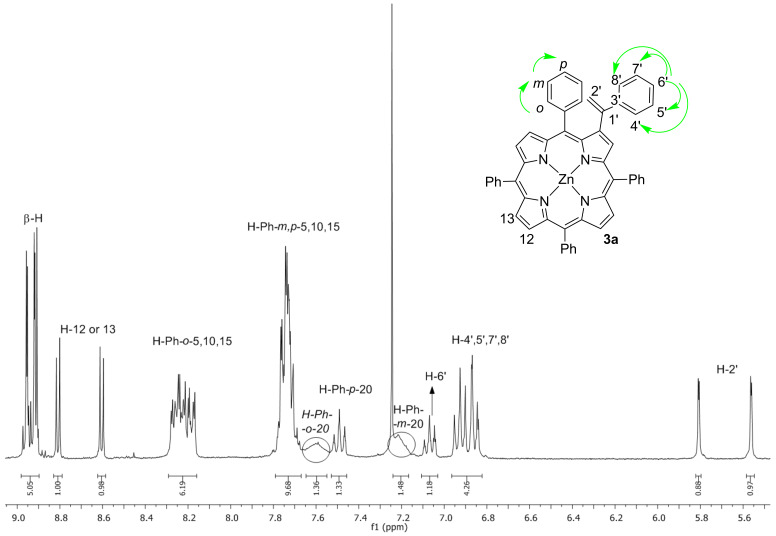
^1^H NMR of β-alkenylporphyrin derivative **3a**. Green arrows illustrate principal COSY correlations.

As a representative example, Figure 1 shows the ^1^H NMR spectrum of the β-vinylporphyrin **3a** and the most important COSY correlations. The spectrum is consistent with a β-substituted porphyrin structure showing a multiplet between 8.98–8.90 ppm which is assigned to the resonance of five β-pyrrolic protons and an AB system at 8.81 and 8.60 ppm (*J* = 4.7 Hz) due to the β-pyrrolic protons H-12 and H-13. The resonances of the *ortho* protons (H-*o*-Ph) and of the *meta* and *para* protons (H-*m,p*-Ph) of the C5, C10 and C15 phenyl groups appear as two multiplets at 7.79–7.67 ppm and 7.24–7.17 ppm, respectively. Due to the lack of rotation of the phenyl group at the C20 position, its H-*o*-Ph protons appear as a broad signal at 7.57 ppm. Through COSY correlations it is possible to identify the H-*m*-Ph of the same group as a broad signal at 7.20 ppm and the H-*p*-Ph as a triplet at 7.49 ppm (*J* = 7.5 Hz). The resonances of the protons H-4’, 5’, 7’, 8’ of the extra Ph group at the β-pyrrolic position appear as a multiplet at 6.97–6.83 ppm. The correlation observed in the COSY spectrum between this multiplet and the triplet at 7.07 ppm (*J* = 7.1 Hz) allowed to attribute unequivocally this resonance to H-6’. Finally, the resonances of the two germinal protons H-2’ of the olefin unit appear as two doublets (*J* = 1.3 Hz) at 5.81 and 5.56 ppm. The value of the coupling constant and the unique correlation of these two protons with a single carbon (115.5 ppm) observed in HSQC spectra corroborates the proposed structure. The HRMS–ESI^+^ spectrum of derivative **3a** shows a molecular ion at the *m*/*z* value 779.2135 corresponding to [M + H]^+^ which confirms also the success of the palladium cross-coupling strategy.

For derivative **3b** and considering only the resonances due to the extra group introduced at the β-pyrrolic position, the resonance of the two protons H-2’ appears as two doublets at 5.78 and 5.60 ppm (*J* = 0.9 Hz). Once again, the value of the coupling constant and the unique correlation of these protons with a single carbon (115.9 ppm) observed in the HSQC spectra validates that H-2’ are geminal protons. The two doublets at 7.04 and 6.71 ppm (*J* = 8.8 Hz) are related to the resonance of H-4’,8’ and H-5’,7’ of the bromophenyl group, respectively. The same profile is observed in ^1^H NMR of derivative **3c**, where the resonance of H-2’ appears as two doublets at 5.67 and 5.45 ppm (*J* = 1.0 Hz) and H-4’,8’ and H-5’,7’ appear as two doublets at 6.76 and 6.42 ppm (*J* = 8.9 Hz), respectively. It is also possible to observe a singlet at 3.61 ppm related to the resonance of the protons of the methoxy group.

The HRMS–ESI^+^ of β-vinylporphyrin derivatives **3b** and **3c** show molecular ions at the *m/z* values of 856.1160 and 808.2174 (M^+•^) respectively, confirming the molecular formula of these derivatives.

All the new porphyrins **3a–c** were also characterized by UV–vis spectroscopy, showing the typical profile of the Zn(II) complex of *meso*-tetraarylporphyrins, with a Soret band at ca. 420 nm and two Q bands between 549 and 586 nm (See Supporting Information File 1).

## Conclusion

In summary, we have reported the efficient synthesis of new β-alkenyl-type porphyrin derivatives through Pd-catalyzed cross-coupling reaction of β-bromoporphyrinatozinc(II) complex with *N*-tosylhydrazones. The best conditions for the reaction of 2-bromo-5,10,15,20-tetraphenylporphyrinatozinc(II) (**1**) and *N*-tosylhydrazone derivatives **2a–c** gave rise to the new β-alkenylporphyrin derivatives **3a–c** in excellent yields (between 86–89%). This is the first report using this methodology in the functionalization of tetrapyrrolic macrocycles. The new compounds that can be achieved by this approach can be excellent templates for further functionalization and also might became compounds with potential important biological activities. This new methodology can be used to have access to a significant number of new porphyrin derivatives and certainly future work will demonstrate this fact.

## Experimental

### Materials and methods

2-Bromo-5,10,15,20-tetraphenylporphyrinatozinc(II) (**1**) and *N*-tosylhydrazone derivatives **2a–c** were prepared according to literature procedures [15–16]. ^1^H and ^13^C NMR spectra were recorded on a Bruker Avance 300 spectrometer at 300.13 and 75.47 MHz and on a Bruker Avance 500 spectrometer at 500.12 and 125.77 MHz. CDCl_3_ was used as solvent and tetramethylsilane (TMS) as internal reference. Chemical shifts are expressed in δ (ppm) and the coupling constants (*J*) are expressed in Hertz. Mass spectra HRMS were recorded on a LTQ Orbitrap XL mass spectrometer (Thermo Fischer Scientific) using CHCl_3_ as solvent. For the reactions under MW irradiation a CEM Microwave Synthesis System Discover SP was used, under constant magnetic stirring (controlled by the microwave equipment) by employing an irradiation power of 200 W till the reaction temperature (120 °C) was achieved. The UV–vis spectra were recorded on an UV-2501 PC Shimadzu spectrophotometer using CHCl_3_ as solvent. Flash chromatography was carried out using silica gel (230–400 mesh) and preparative thin-layer chromatography was carried out on 20 × 20 cm glass plates coated with silica gel (1 mm thick). The reactions were routinely monitored by thin-layer chromatography (TLC) on silica gel precoated F_254_ Merck plates.

### General procedures for synthesis of β-alkenyl porphyrin derivatives **3a–c**

#### Using Pd(PPh_3_)_4_, XPhos and KO*t*-Bu as catalytic system under conventional heating

2-Bromo-5,10,15,20-tetraphenylporphyrinatozinc(II) (**1**, 20 mg, 26.4 µmol), Pd(PPh_3_)_4_ (2 mol %), XPhos ligand (10 mol %, 2.64 µmol, 1.2 mg), *N*-tosylhydrazones **2a–c** (1 equiv, 26.4 µmol), KO*t-*Bu (2.5 equiv, 66 µmol, 7.4 mg) were placed in a 50 mL Schlenk tube and dissolved in 5 mL of toluene and 5 mL of dioxane. The solvent was degassed by repeated sonication for 1–2 min under reduced pressure. The reaction mixture was stirred during 24 h at 120 °C. After cooling, the reactions were quenched with water, extracted with toluene and each organic layer was washed with water, dried in Na_2_SO_4_ and concentrated under reduced pressure. Each crude mixture was purified by preparative TLC using hexane/CH_2_Cl_2_ (1:1) as the eluent in each case.

#### Using Pd(PPh_3_)_4_, Et_4_NBr and Cs_2_CO_3_ as catalytic system under conventional heating

In a two neck round-bottom flask under nitrogen atmosphere were placed 2-bromo-5,10,15,20-tetraphenylporphyrinatozinc(II) (**1**, 20 mg, 26.4 µmol) and the *N*-tosylhydrazones **2a–c** (2 equiv, 52.8 µmol). These solids were maintained under nitrogen flux during 5 minutes. Pd(PPh_3_)_4_ (5 mol %, 1.32 µmol, 1.5 mg), Cs_2_CO_3_ (3 equiv, 79.2 µmol, 27.9 mg) and Et_4_NBr (20 mol %, 5.28 µmol, 1.10 mg) were then added and the obtained mixture in each case was dissolved in toluene (1 mL) and DMF (0.5 mL). The reactions were maintained at 120 °C for 4 h. After cooling, the reactions were quenched with water, extracted with toluene and the organic layers were washed with water, dried in Na_2_SO_4_ and concentrated under reduced pressure. The crude mixtures were purified by preparative TLC hexane/CH_2_Cl_2_ (1:1) as the eluents.

#### Using Pd(PPh_3_)_4_, Et_4_NBr and Cs_2_CO_3_ as catalytic system under MW irradiation

In a MW reactor were placed 2-bromo-5,10,15,20-tetraphenylporphyrinatozinc(II) (**1**, 20 mg, 26.4 µmol), *N*-tosylhydrazones **2a–c** (2 equiv, 52.8 µmol), Pd(PPh_3_)_4_ (5 mol %, 1.32 µmol, 1.5 mg), Cs_2_CO_3_ (3 equiv, 79.2 µmol, 27.9 mg) and Et_4_NBr (20 mol %, 5.28 µmol, 1.10 mg). The obtained mixtures were dissolved in toluene (1 mL) and DMF (0.5 mL). The reactions were irradiated under MW radiation under 200 W for 5 minutes at 120 °C. After cooling, the reactions were quenched with water, extracted with toluene and each organic layer was washed with water, dried in Na_2_SO_4_ and concentrated under reduced pressure. The final reaction mixtures were purified by preparative TLC using hexane/CH_2_Cl_2_ (1:1) as the eluent in each case.

### Structural characterization of β-alkenylporphyrin derivatives **3a–c**

#### 2-(1-Phenylethenyl)-5,10,15,20-tetraphenylporphyrinatozinc(II) (**3a**)


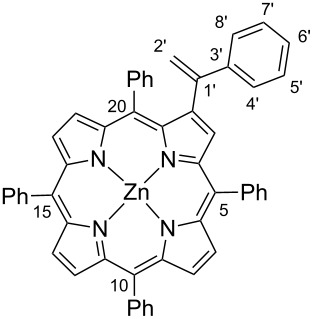


^1^H NMR (300 MHz, CDCl_3_) δ 8.98–8.90 (m, 5H, β-H), 8.81 and 8.60 (AB system, *J* = 4.7 Hz, 2H, H-12 and H-13), 8.29–8.16 (m, 6H, H-Ph-*o-*5,10,15), 7.79–7.67 (m, 9H, H-Ph-*m,p-*5,10,15), 7.57 (bs, 2H, H-Ph-*o-*20), 7.49 (t, *J* = 7.5 Hz, 1H, H-Ph-*p-*20), 7.20 (bs, 2H, H-Ph-*m-*20), 7.07 (t, *J* = 7.1Hz, 1H, H-6’), 6.97–6.83 (m, 4H, H-4’,5’,7’,8’), 5.81 (d, *J* = 1.3 Hz, 1H, H-2’), 5.56 (d, *J* = 1.3 Hz, 1H, H-2’); ^13^C NMR (126 MHz, CDCl_3_) δ 151.4, 150.4, 150.3, 150.2, 150.2, 150.1, 150.0, 148.1, 147.2, 146.2, 145.6, 142.9, 142.7, 141.2, 140.8, 136.1 (β-C), 135.5 (C-Ph-*o-*5,10,15), 134.5 (C-Ph-*o-*5,10,15), 134.46 (C-Ph-*o-*5,10,15), 134.41 (C-Ph-*o-*5,10,15), 132.7 (C-12 or C-13), 132.1 (β-C), 132.0 (β-C), 131.99 (β-C), 131.91 (β-C), 131.4 (C-12 or C-13), 127.5, 127.5, 127.4, 127.1, 127.0, 126.6, 126.56, 126.1, 125.5 (C-Ph-*m-*20), 122.3, 121.4, 121.0, 120.4, 115.5 (C-2’); UV–vis (CH_2_Cl_2_) λ_max_ (log ε): 422 (5.30) 549 (3.95) 586 (3.15) nm; HRMS–ESI: *m*/*z*: [M + H]^+^ calcd for C_52_H_35_N_4_Zn^+^, 779.2147; found, 779.2135.

#### 2-[1-(4-Bromophenyl)ethenyl]-5,10,15,20-tetraphenylporphyrinatozinc(II) (**3b**)


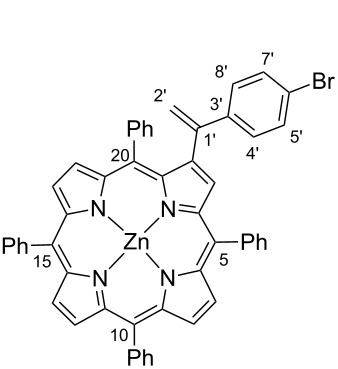


^1^H NMR (300 MHz, CDCl_3_) δ 9.02–8.85 (m, 5H, β-H), 8.81 and 8.60 (AB system, *J* = 4.7 Hz, 2H, H-12 and H-13), 8.36–8.01 (m, 6H, H-Ph-*o-*5,10,15), 7.76–7.71 (m, 9H, H-Ph-*m,p-*5,10,15), 7.51 (t, *J* = 7.6 Hz, 1H, H-Ph-*p-*20), 7.53–7.48 (m, *2*H, H-Ph-*o-*20), 7.40–7.37 (m, 2H, H-Ph-*m-*20), 7.04 (d, *J* = 8.8 Hz, 2H, H-4’,8’), 6.71 (d, *J* = 8.8 Hz, 2H, H-5’,7’), 5.78 (s, 1H, H-2’), 5.60 (s, 1H, H-2’); ^13^C NMR (75 MHz, CDCl_3_) δ 151.4, 150.5, 150.3, 150.3, 150.25, 150.10, 147.9, 146.8, 145.4, 144.8, 142.9, 142.8, 142.72, 141.2, 139.7, 136.1, 134.5 (C-Ph-*o*-5,10,15), 134.4 (C-Ph-*o*-5,10,15), 132.8 (C-Ph-*o*-5,10,15), 132.2 (C-Ph-*o*-5,10,15), 132.1 (C-12 or C-13), 132.0 (β-C), 131.9 (β-C), 131.5 (β-C), 131.2 (β-C), 130.5 (β-C), 129.8 (C-12 or C-13), 131.4, 131.3, 131.2, 130.4 (C-4’,8’), 129.7, 129.4, 127.7 (C-5’,7’), 127.5, 127.2, 126.6, 126.7, 122.0, 121.5, 121.1, 121.0, 120.4, 116.0 (C-2’); UV–vis (CH_2_Cl_2_) λ_max_ (log ε): 422 (5.22) 549 (3.89) 586 (3.21) nm; HRMS–ESI: *m*/*z*: M^+•^ calcd for C_52_H_33_BrN_4_Zn, 856.1174; found, 856.1160.

#### 2-[1-(4-Methoxyphenyl)ethenyl]-5,10,15,20-tetraphenylporphyrinatozinc(II) (**3c**)


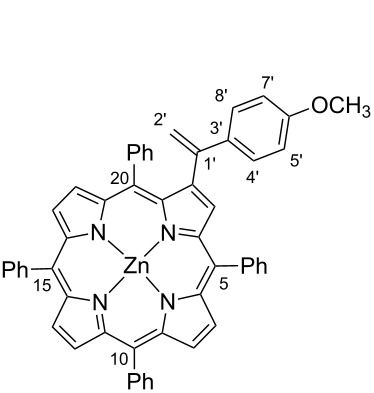


^1^H NMR (300 MHz, CDCl_3_) δ 8.97–8.88 (m, 5H, β-H), 8.81 and 8.61 (AB system, *J* = 4.7 Hz, 2H, H-12 and H-13), 8.28–8.16 (m, 6H, H-Ph-*o-*5,10,15), 8.06 (bs, 2H, H-Ph-*o*-20) 7.78–7.67 (m, 11H, H-Ph-*m,p-*5,10,15 + H-Ph-*m*-20), 7.48 (t, *J* = 7.5 Hz, 1H, H-Ph-*p-*20), 6.76 (d, *J* = 8.9 Hz, 2H, H-4’,8’), 6.42 (d, *J* = 8.9 Hz, 2H, H-5’,7’), 5.67 (d, *J* = 1.0 Hz, 1H, H-2’), 5.45 (d, *J* = 1.0 Hz, 1H, H-2’), 3.61 (s, 3H, OCH_3_); ^13^C NMR (126 MHz, CDCl_3_) δ 158.9, 151.4, 150.39, 150.3, 150.2, 150.1, 149.9, 148.2, 147.3, 146.5, 145.0, 142.9, 142.8, 141.2, 135.9 (β-C), 134.5, 134.48, 134.43, 134.0, 132.7 (C-12 or C-13), 132.1 (β-C), 132.0 (β-C), 131.9 (β-C), 131.8 (β-C), 131.4 (C-12 or C-13), 127.53, 127.50 (C-4’,8’), 127.3, 127.0, 126.6, 126.5, 125.5, 122.3, 121.4, 121.0, 120.3, 113.8 (C-2’), 112.7 (C-5’,7’), 55.2 (O*C*H_3_); UV–vis (CH_2_Cl_2_) λ_max_ (log ε): 421 (5.90) 550 (4.19) 587 (3.46) nm. HRMS–ESI: *m*/*z*: M^+•^ calcd for C_53_H_36_N_4_OZn, 808.2175; found, 808.2174.

## Supporting Information

File 1 Copies of NMR, HRMS(ESI) and UV–vis spectra of the new derivatives.
