# Supplementary material for: Efficient access to β-vinylporphyrin derivatives via palladium cross coupling of β-bromoporphyrins with N-tosylhydrazones
Source: Beilstein J Org Chem. 2017 Jan 30;13:195–202. doi: 10.3762/bjoc.13.22 (PMC5301804; doi:10.3762/bjoc.13.22)
Supplement: File 1 — Copies of NMR, HRMS(ESI) and UV–vis spectra of the new derivatives. [file Beilstein_J_Org_Chem-13-195-s001.pdf]

Supporting Information

for

Efficient access to β-vinylporphyrin derivatives via palladium cross coupling of β-bromoporphyrins with *N*-tosylhydrazones

Vinicius R. Campos[1,2], Ana T. P. C. Gomes *[2], Anna C. Cunha[1]*, Maria da Graça P. M. S. Neves[2], Vitor F. Ferreira[1] and José A. S. Cavaleiro*[2]

Address: [1]Departamento de Química Orgânica, Instituto de Química, Universidade Federal Fluminense, 24020-150 Niterói, RJ, Brazil and [2]QOPNA and Department of Chemistry, University of Aveiro, 3810-193 Aveiro, Portugal.

Email: Ana T. Peixoto C. Gomes - ana.peixoto@ua.pt; Anna C. Cunha - annac@vm.uff.br; José A. S. Cavaleiro - jcavaleiro@ua.pt

Dedicated to the memory of Professor José Barluenga, Oviedo University, Oviedo, Spain.

Copies of NMR, HRMS(ESI) and UV–vis spectra of the new derivatives.

Analytical data

Structural characterization of β-alkenyl porphyrin derivatives **3a–c**

^{13}C NMR spectrum of β-alkenyl porphyrin derivative **3a** in CDCl$_3$……………………………………………………………………….....................**S4**

COSY spectrum of β-alkenyl porphyrin derivative **3a** in CDCl$_3$…………………………………………………………..……………..**S4**

HSQC spectrum of β-alkenyl porphyrin derivative **3a** in CDCl$_3$………………………………………………………………………………**S5**

HMBC spectrum of β-alkenyl porphyrin derivative **3a** in CDCl$_3$…………………………………………………………..…………**S5**

HR(ESI$^+$) spectrum of β-alkenyl porphyrin derivative **3a**…………………………………………………………..…………………**S6**

^{1}H NMR spectrum of β-alkenyl porphyrin derivative **3b** in CDCl$_3$……………………………………………………………………………**S6**

^{13}C NMR spectrum of β-alkenyl porphyrin derivative **3b** in CDCl$_3$……………………………………………………………………………**S7**

COSY spectrum of β-alkenyl porphyrin derivative **3b** in CDCl$_3$…………………………………………………………………………..**S7**

HSQC spectrum of β-alkenyl porphyrin derivative **3b** in CDCl$_3$……………………………………………………………………..…**S8**

HMBC spectrum of β-alkenyl porphyrin derivative **3b** in CDCl$_3$…………………………………………………………………………..**S8**

HR(ESI$^+$) spectrum of β-alkenyl porphyrin derivative **3b**…………………...**S9**

^{1}H NMR spectrum of β-alkenyl porphyrin derivative **3c** in CDCl$_3$…………………………………………………………………………..**S9**

^{13}C NMR spectrum of β-alkenyl porphyrin derivative **3c** in

$CDCl_3$……………………………………………………………………..….**S10**

COSY spectrum of β-alkenyl porphyrin derivative **3c** in

$CDCl_3$………………………………………………………………………...**S10**

HSQC spectrum of β-alkenyl porphyrin derivative **3c** in

$CDCl_3$………………………………………………………………………...**S11**

HMBC spectrum of β-alkenyl porphyrin derivative **3c** in

$CDCl_3$………………………………………………………………………...**S11**

HR(ESI$^+$) spectrum of β-alkenyl porphyrin derivative **3c**…......................**S12**

Normalized UV–vis spectra of in CH_2Cl_2 of β-alkenyl porphyrin derivatives

3a–c……………………………………………………………………………....**S12**

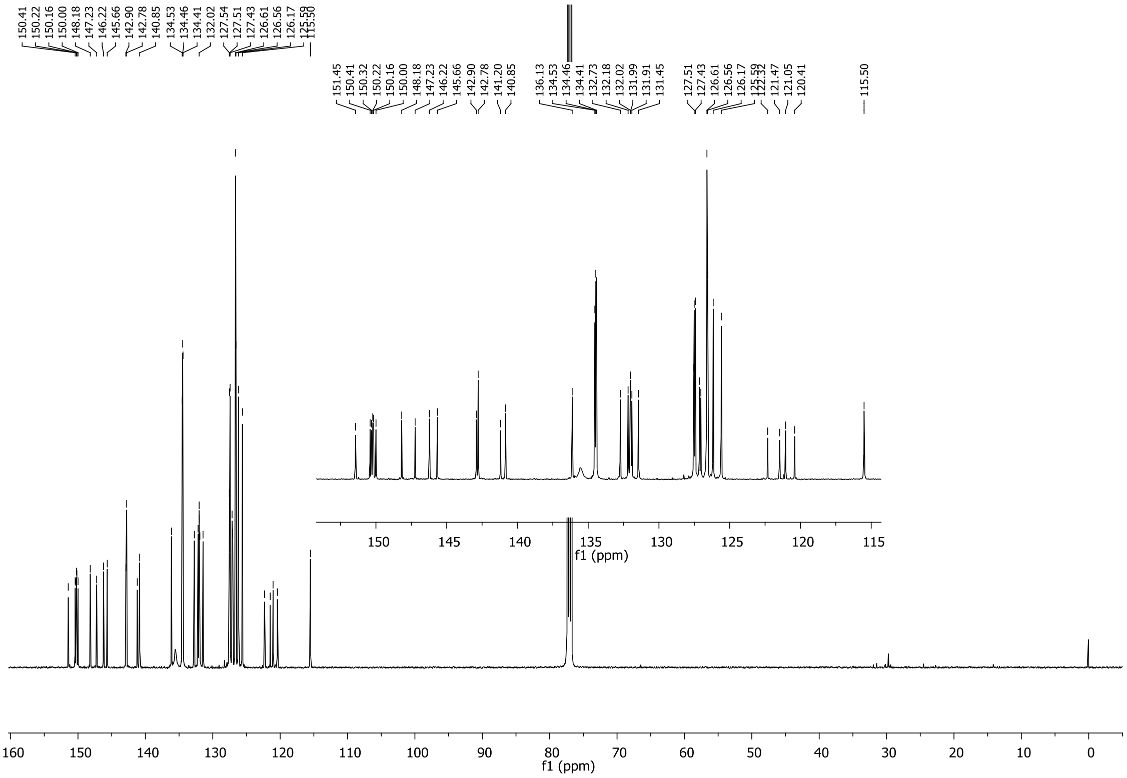

Figure S1 - ^{13}C NMR spectrum of β-alkenyl porphyrin derivative **3a** in $CDCl_3$

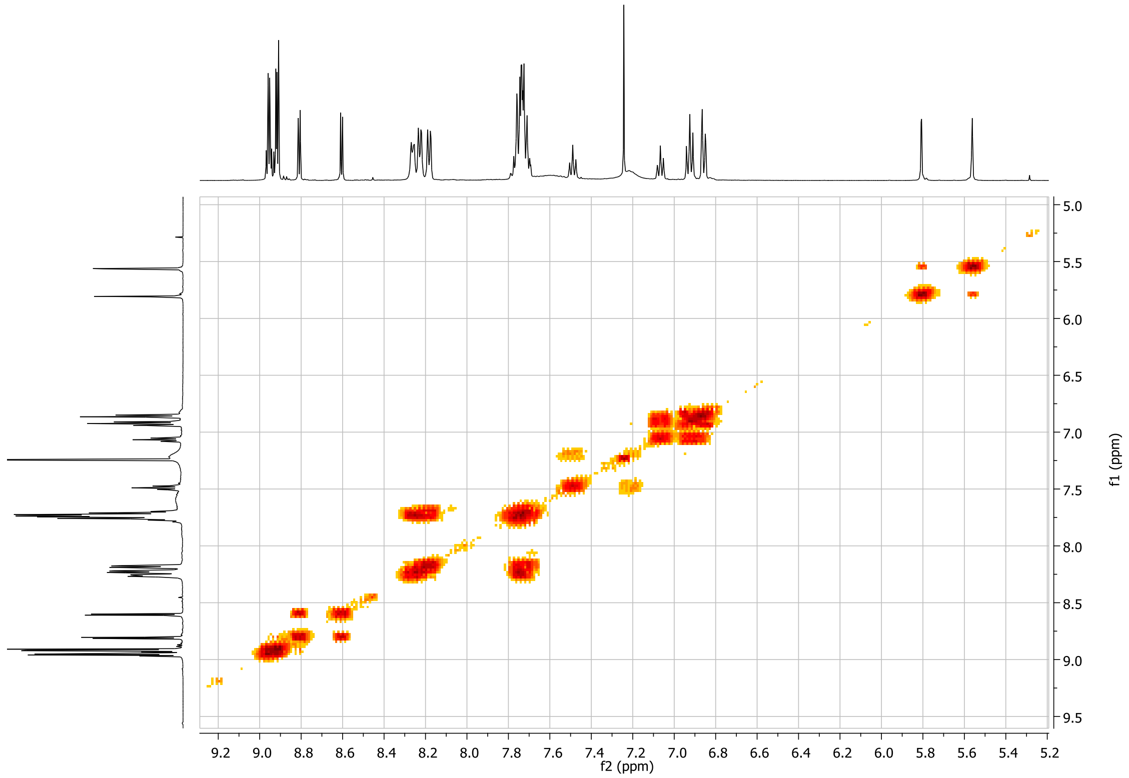

Figure S2 - COSY spectrum of β-alkenyl porphyrin derivative **3a** in $CDCl_3$

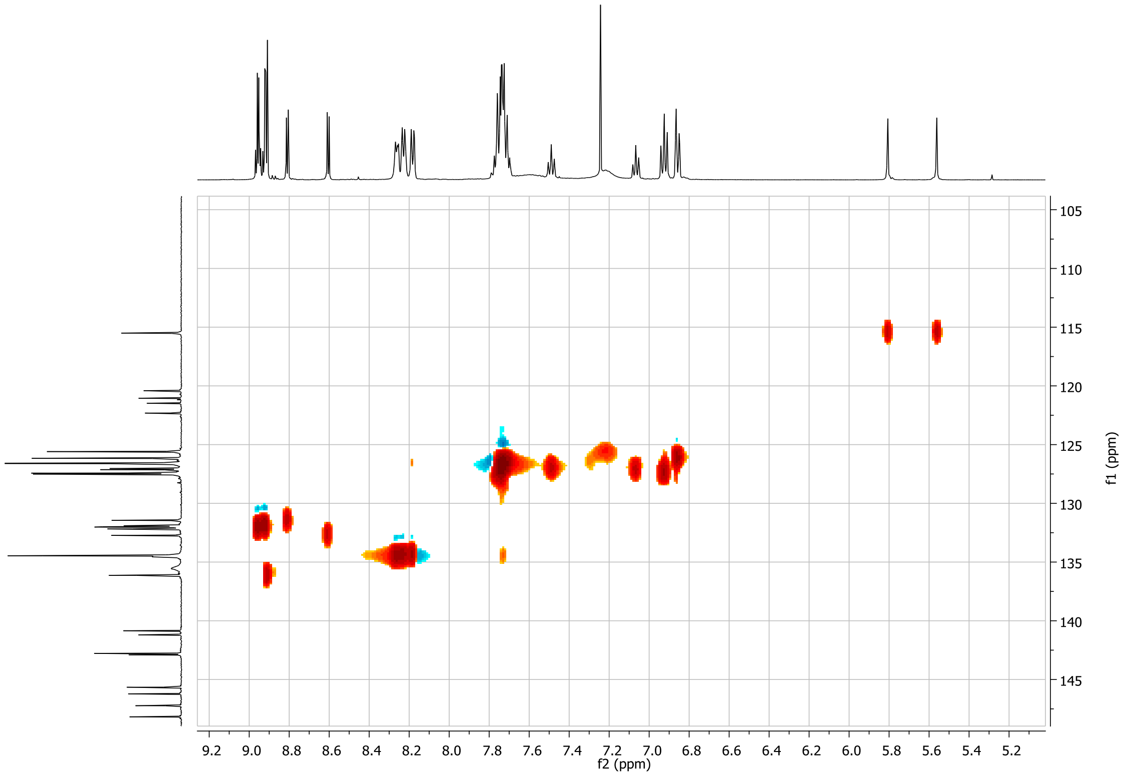

Figure S3 - HSQC spectrum of β-alkenyl porphyrin derivative **3a** in $CDCl_3$

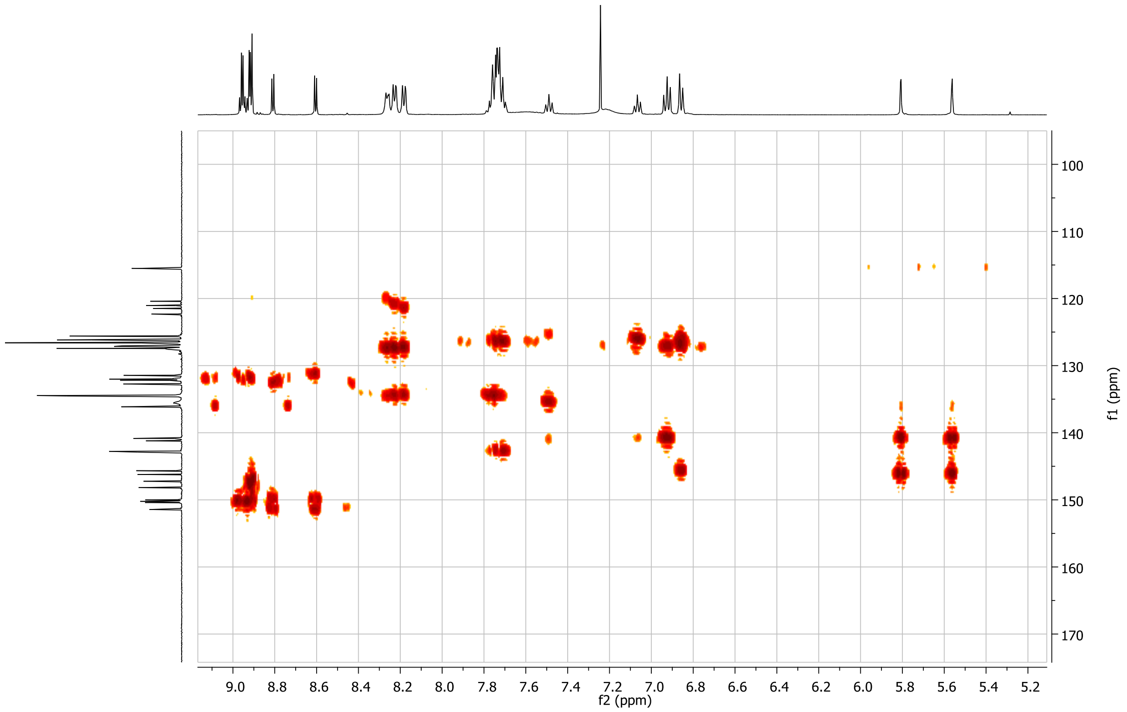

Figure S4 - HMBC spectrum of β-alkenyl porphyrin derivative **3a** in $CDCl_3$

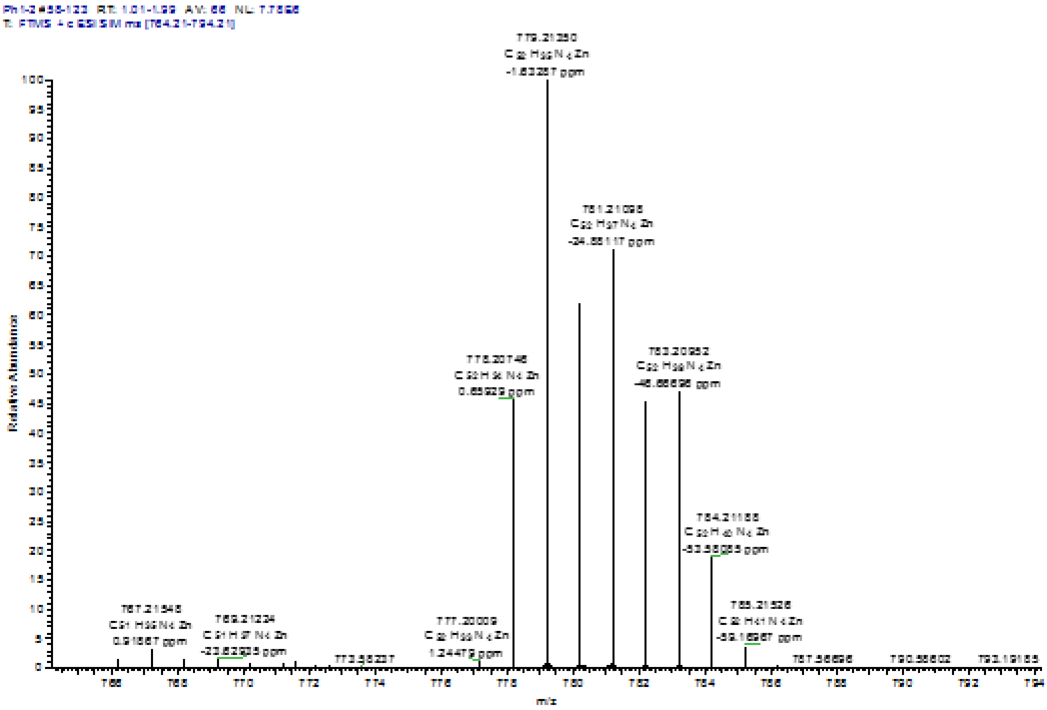

Figure S5 - HR(ESI$^+$) spectrum of β-alkenyl porphyrin derivative **3a**

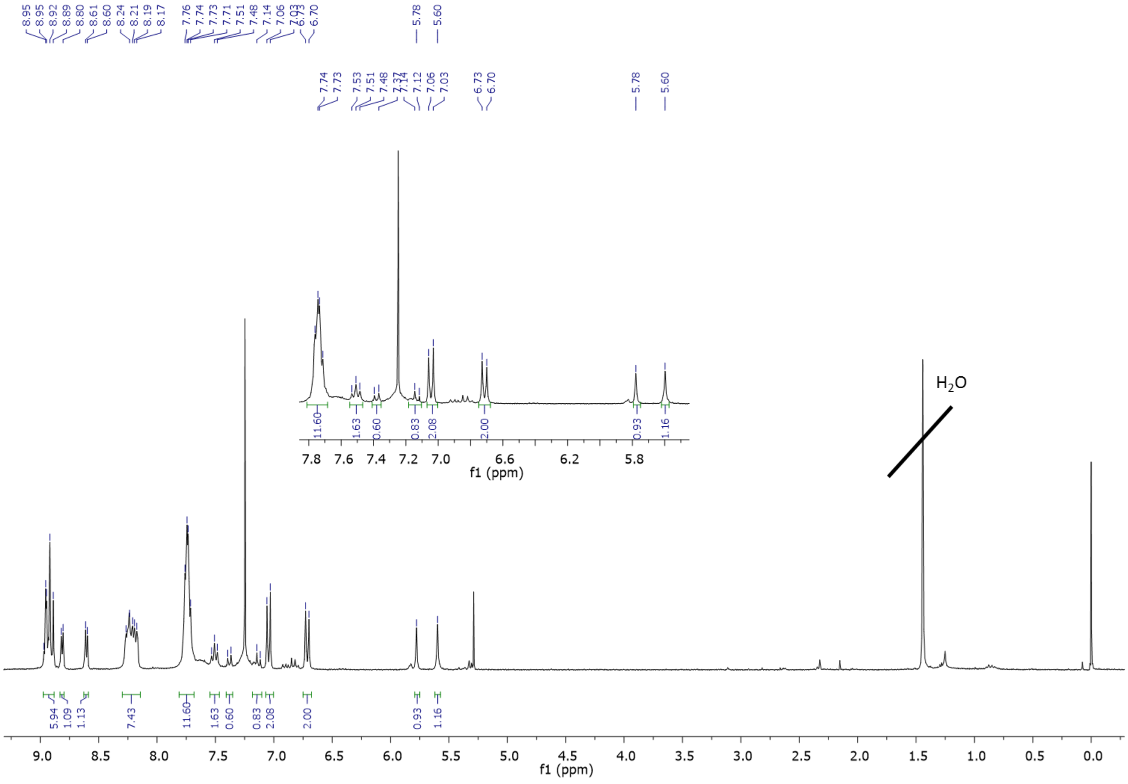

Figure S6 - ^{1}H NMR spectrum of β-alkenyl porphyrin derivative **3b** in $CDCl_3$

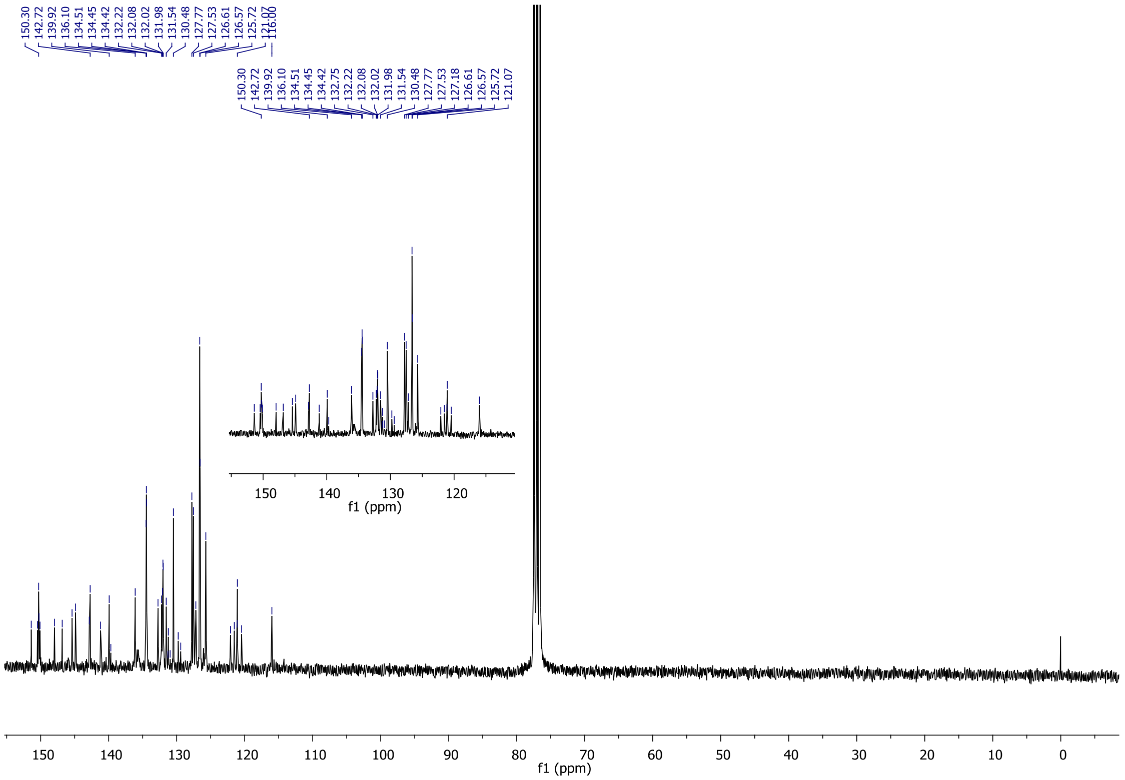

Figure S7 - ^{13}C NMR spectrum of β-alkenyl porphyrin derivative **3b** in $CDCl_3$

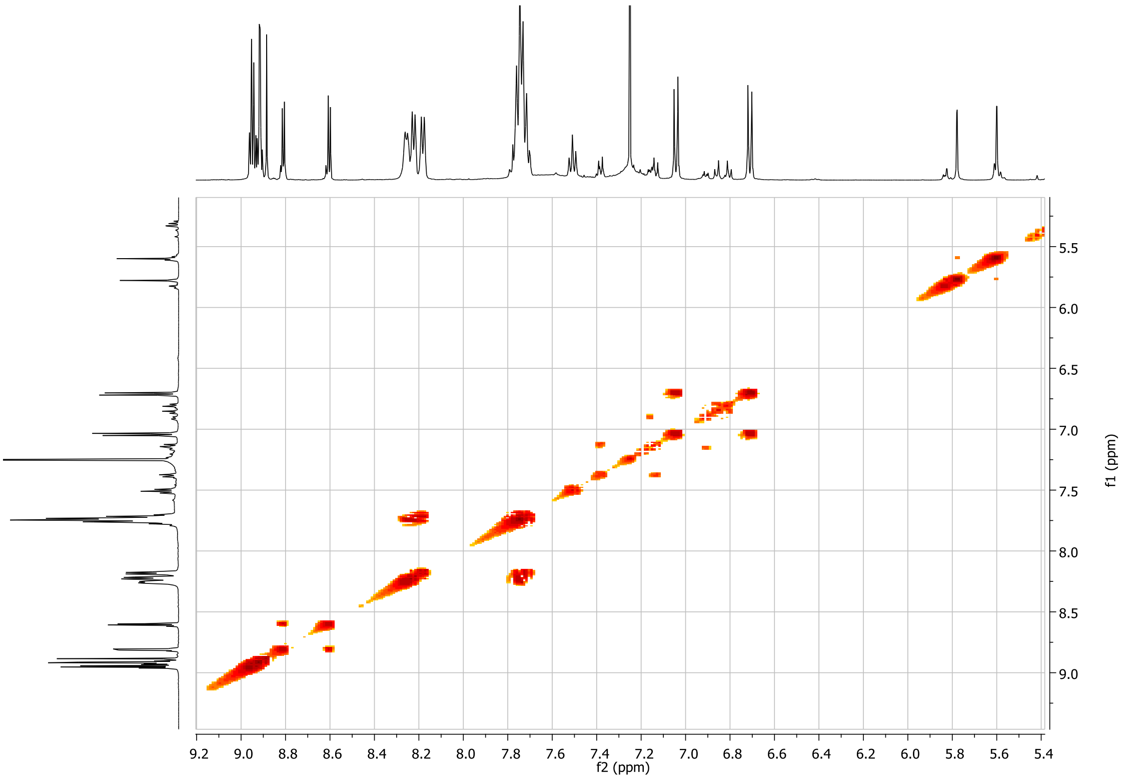

Figure S8 - COSY spectrum of β-alkenyl porphyrin derivative **3b** in $CDCl_3$

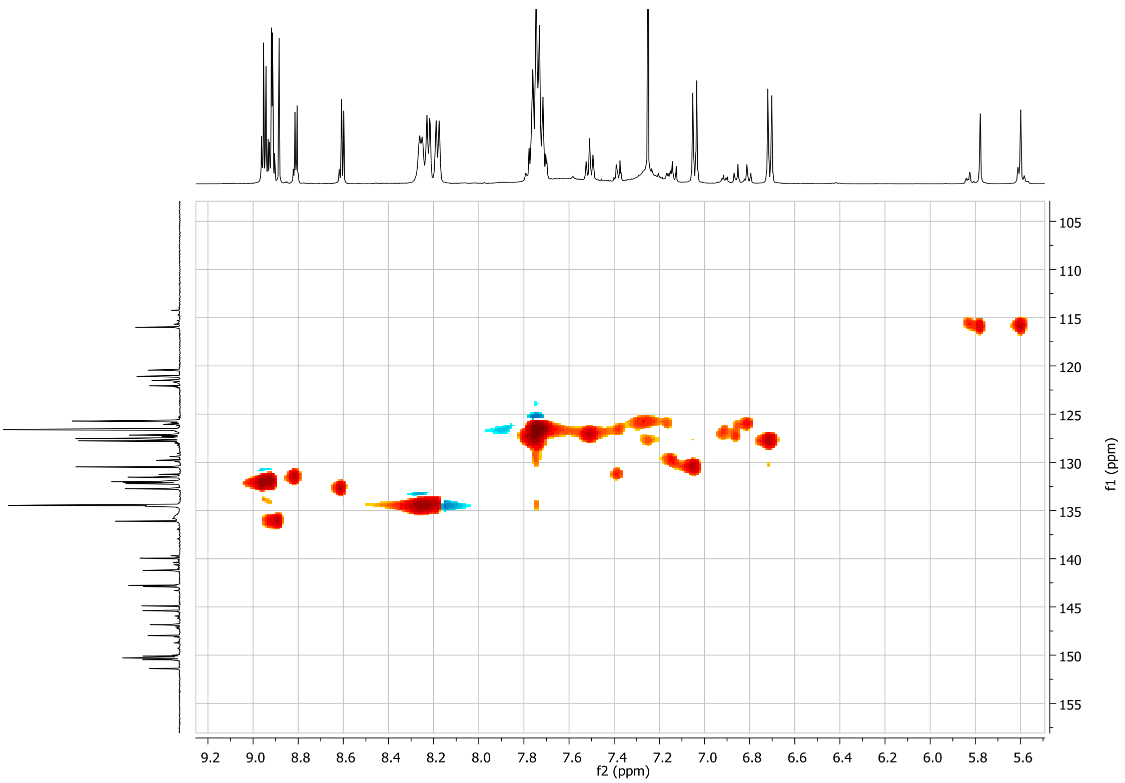

Figure S9- HSQC spectrum of β-alkenyl porphyrin derivative **3b** in $CDCl_3$

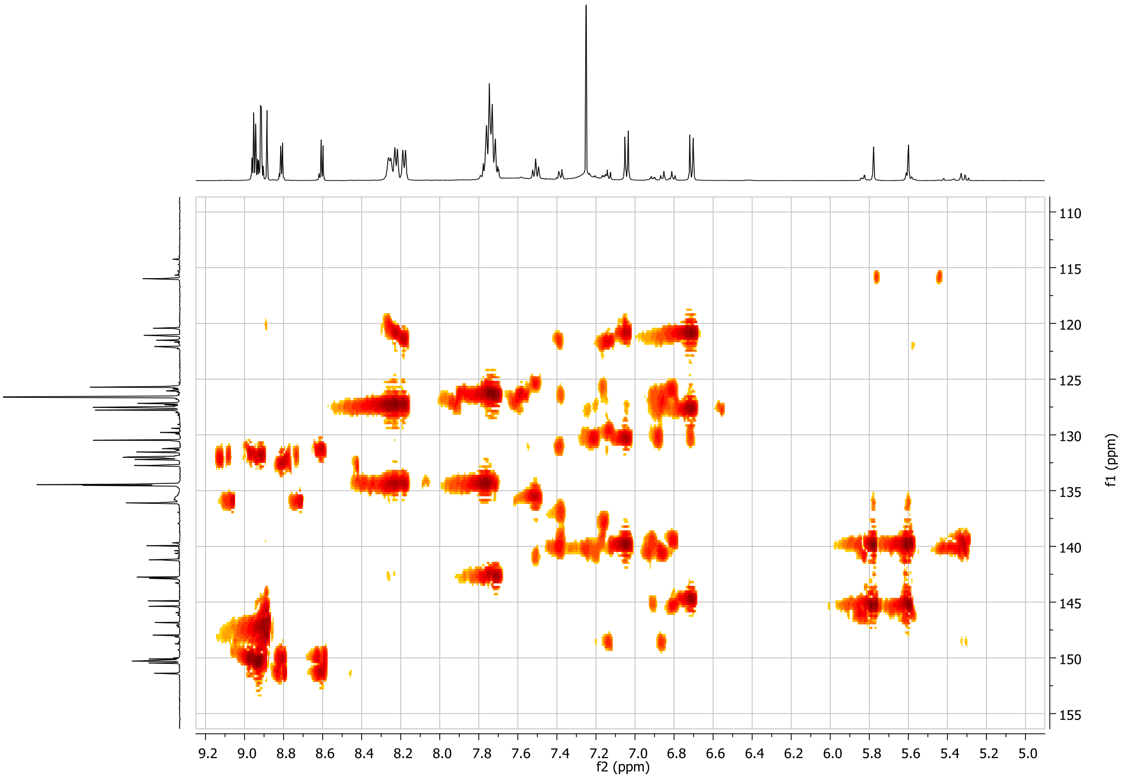

Figure S10- HMBC spectrum of β-alkenyl porphyrin derivative **3b** in $CDCl_3$

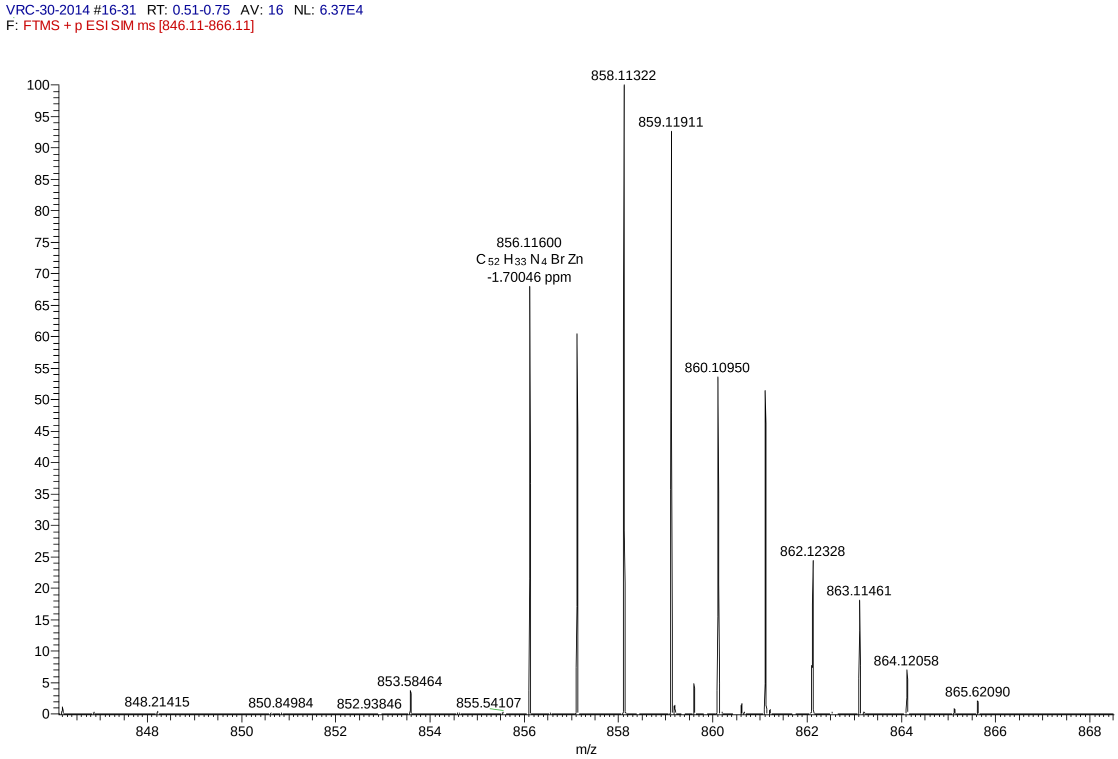

Figure S11 - HR(ESI$^+$) spectrum of β-alkenyl porphyrin derivative **3b**

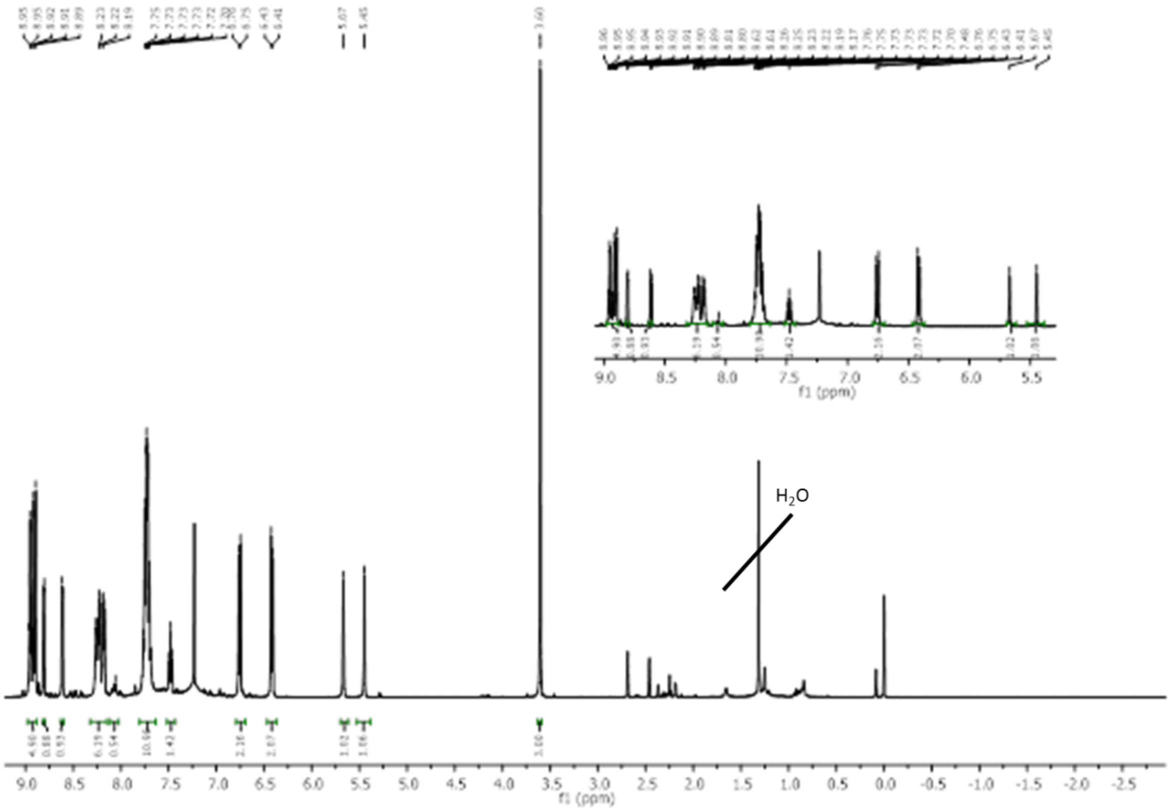

Figure S12 - ^{1}H NMR spectrum of β-alkenyl porphyrin derivative **3c** in $CDCl_3$

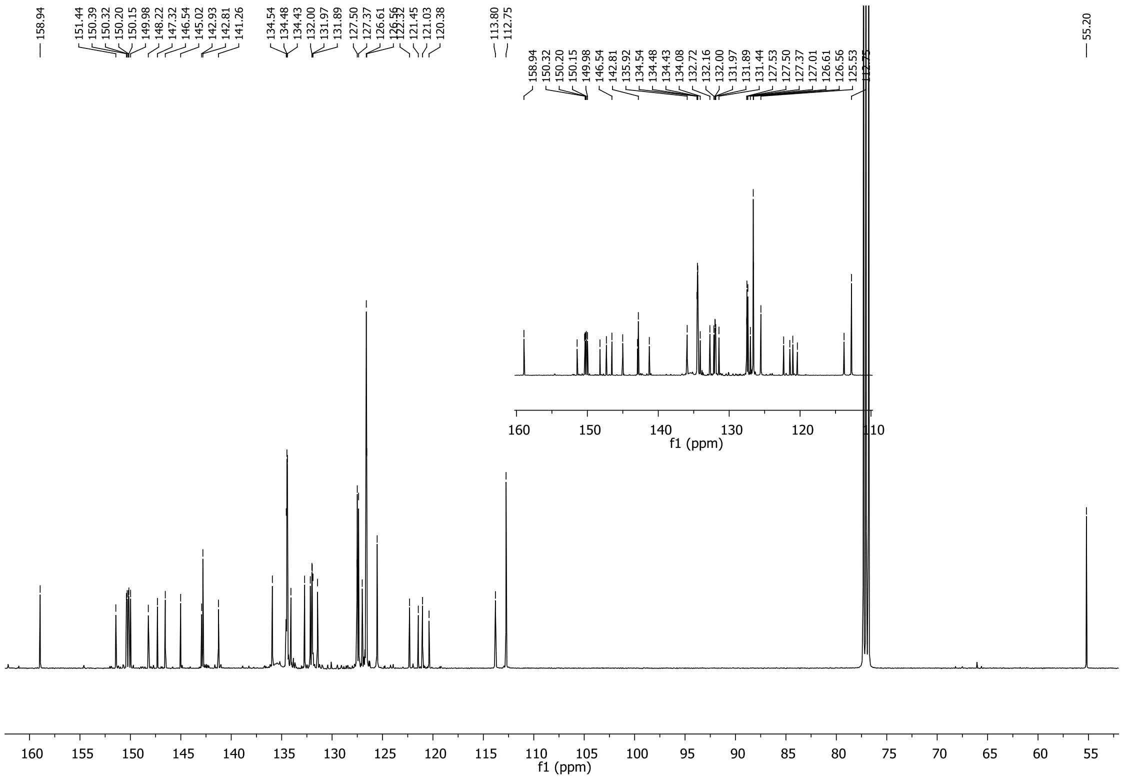

Figure S13 - ^{13}C NMR spectrum of β-alkenyl porphyrin derivative **3c** in $CDCl_3$

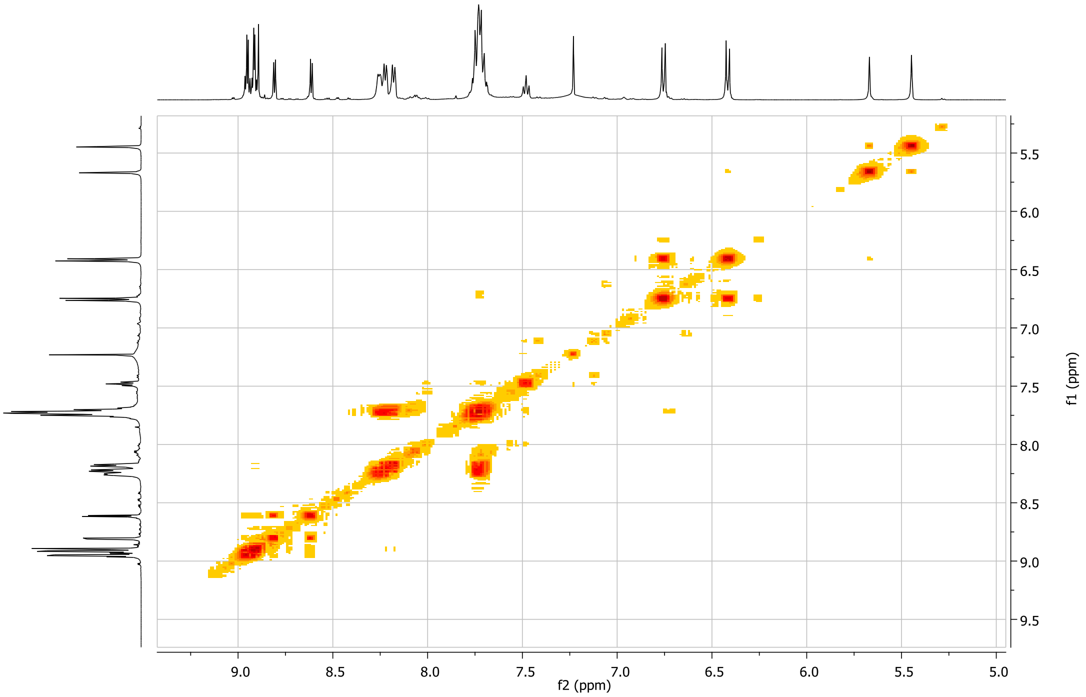

Figure S14 - COSY spectrum of β-alkenyl porphyrin derivative **3c** in $CDCl_3$

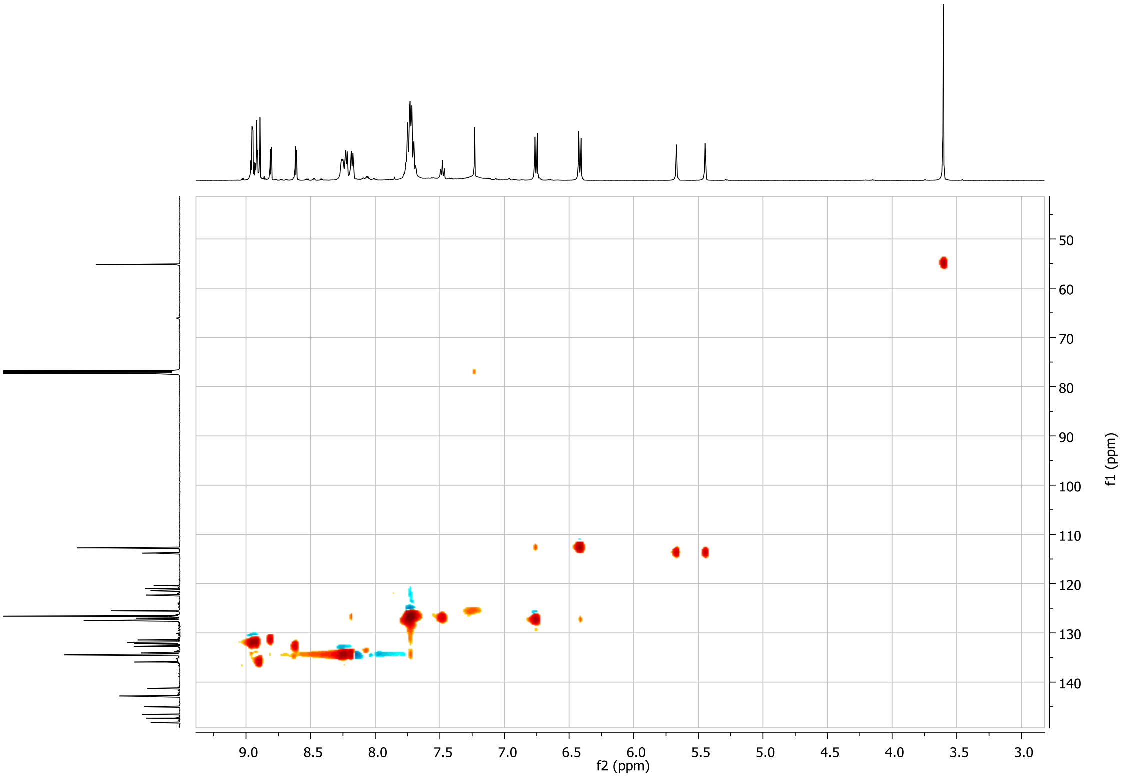

Figure S15 - HSQC spectrum of β-alkenyl porphyrin derivative **3c** in $CDCl_3$

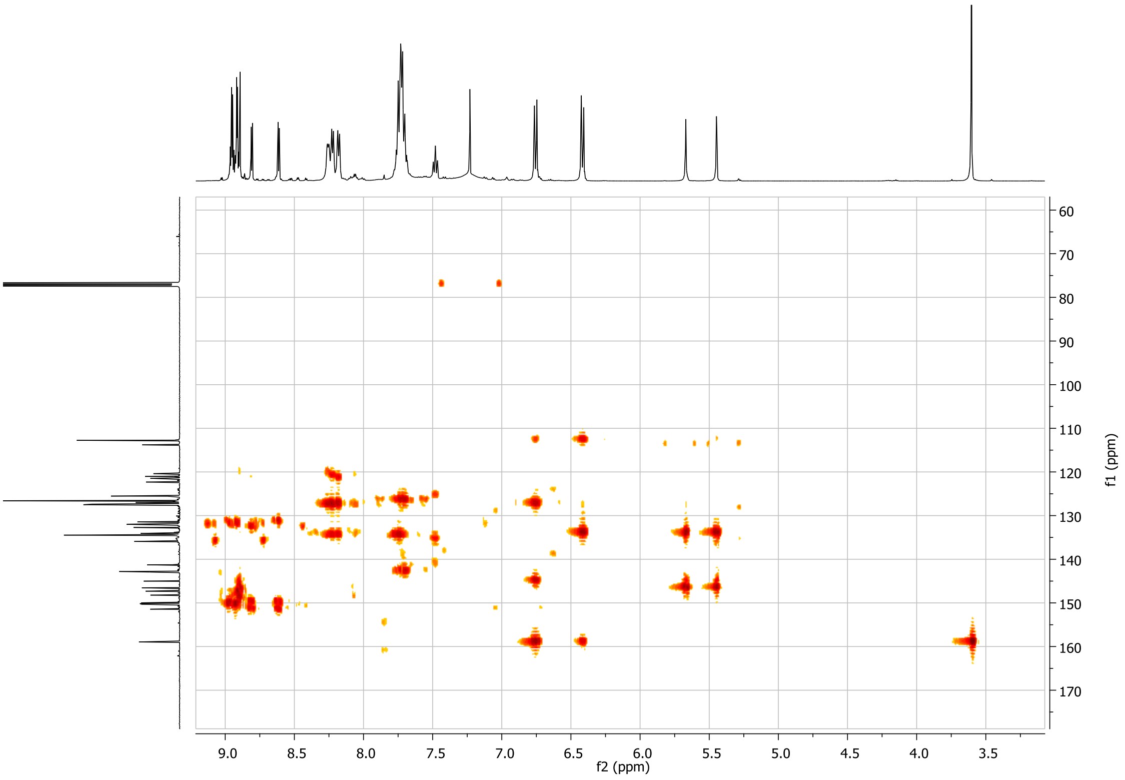

Figure S16 - HMBC spectrum of β-alkenyl porphyrin derivative **3c** in $CDCl_3$

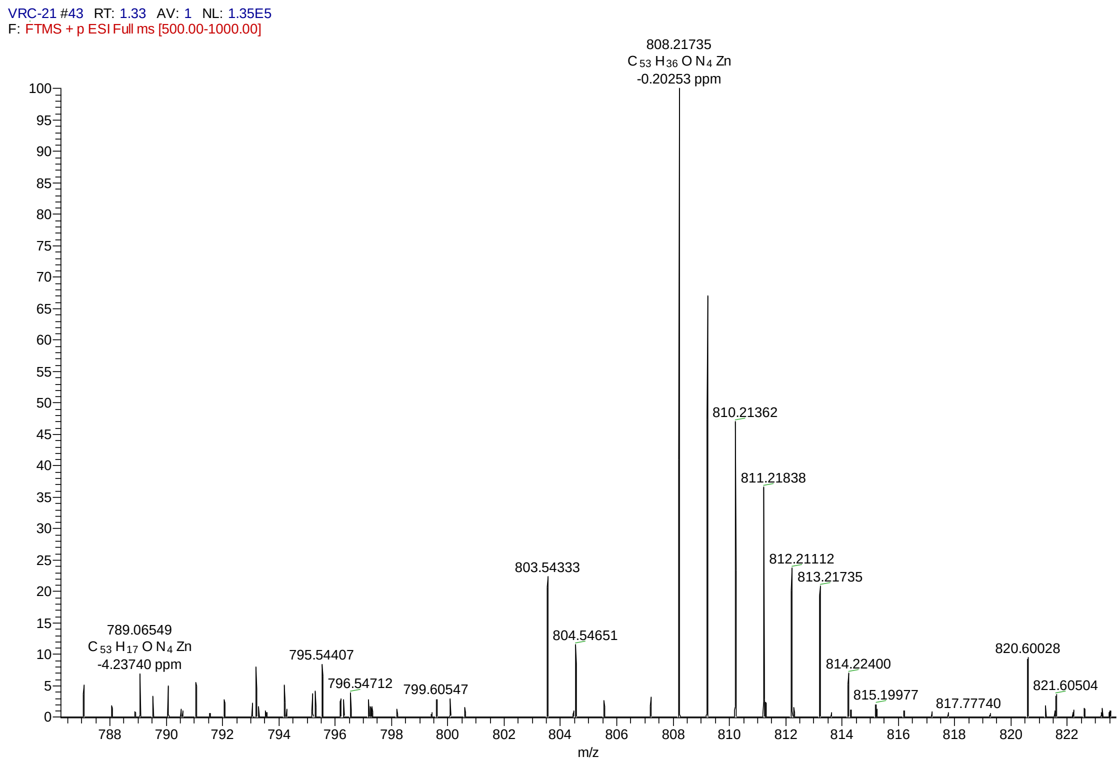

Figure S17 - HR(ESI$^+$) spectrum of β-alkenyl porphyrin derivative **3c**

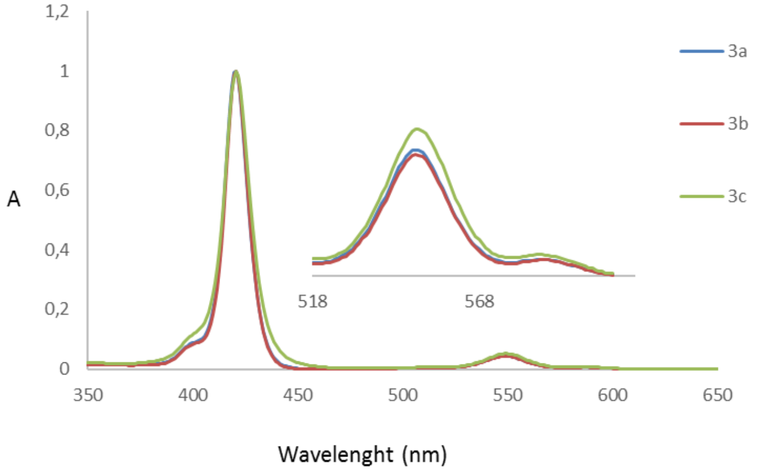

Figure S18 - Normalized UV–vis Spectra of in CH_2Cl_2 of β-alkenyl porphyrin derivatives **3a–c.**
